# Mr. Mayo's Outlines of Human Physiology

**Published:** 1833-07-01

**Authors:** 


**Outlines of Human Physiology.** By *Herbert Mayo*, F.R.S. F.G.S. Professor of Anatomy in King's College, London; Surgeon to the Middlesex Hospital. Formerly Professor of Anatomy and Surgery to the Royal College of Surgeons. The Third Edition, 8vo, pp. 478. London, 1833.

We take shame to ourselves for not having directed the attention of our readers to this really excellent work at an earlier period. As it is not our habit to notice at length elementary productions, we disregarded this amongst the crowd, and were not aware till recently of its many merits. The present edition is so much enlarged, and contains new matter of so interesting a kind that we willingly take the opportunity of devoting some pages to its consideration. The work itself will be chiefly purchased by students; the portions to which we shall apply ourselves will be found to offer subject of reflection to those who are long past the age of pupillage, and thus we think we shall induce some of our elder professional brethren, especially in the country, to make themselves familiar with a work which all should possess, who are anxious to keep pace with the advancing knowledge of the day. Indeed this work is itself no mean evidence of the rapidity of that advance. The edition before us and the first, which was printed but three or four years ago, are almost different works, so great is the addition of facts in the present. That such a treatise should be well appreciated by young men in the present day says much in their favour, and in favour of the intelligence of the age.

The part to which we now beg to call attention is that which is devoted to the consideration of the functions of the nervous system; and we may state at once, that, as we only propose to select such views or statements as may appear novel, or may be interesting on other grounds, we shall not, of course, offer a systematic analysis or criticism.

Mr. Mayo commences with an examination of the mental phenomena in man and animals. "It is difficult," says he, "to disbelieve that a common principle of consciousness exists in both; in the one expanded into a reasonable nature, in the other narrowed and subjected to blind impulse and necessity." In the latter opinion we cannot coincide with Mr. Mayo, but probably an opportunity of investigating this question more fully will present itself farther on. Mr. Mayo makes the following remarks upon sensation and perception.

"When adequate impressions are made upon our organs we are conscious of sensation. When, for example, coloured rays impinge upon the retina, sensations of light are produced in us. *Perception* is a term which in common discourse is used synonymously with sensation: we employ, for instance, indifferently the expressions, to experience sensations of colour, and to perceive colours. But metaphysicians attach different meanings to the words sensation and perception, and use the latter to express the knowledge of the presence and qualities of external objects which follows upon sensation. In order thoroughly to sift this distinction, let us analyse the very complicated impression which is conveyed to the mind through momentary exercise of an organ of sense. I look, for instance, at an object of such dimensions, that a glance serves to satisfy me respecting its nature: the impression which I receive through this experiment is threefold: comprising, 1. Present sensations of colour: 2. A conviction that those sensations are excited by something external: 3. A knowledge of the real size and form and distance of the object which I have seen. The second of these impressions, or the notion which we form of a something external as the cause of sensation, constitutes perception. The third class of impressions that I have described, and which we have learned to associate with the preceding, are our acquired perceptions.

Upon comparing sensation and perception in animals with the like affections in man, it is evident that the former have a partial superiority as to both. The vision of many birds, the sense of smell in birds, and in a still higher degree in many quadrupeds, is more acute than our own. The sense of smell in animals is closely connected with their most powerful instincts. Its existence and its force may be traced even in creatures of a different caste of organization from ourselves. The aversion, for instance, which bees are said to show to particular individuals is probably excited through this sense. How far in man as in animals this sense might come to suggest motives of conduct, is curiously shown in the account by Dugald Stewart of James Mitchell, who had the misfortune to be born deaf and blind, and was thus deprived of the two most important channels through which knowledge is ordinarily received. I may quote the following statement, given upon the authority of Mr. Wardrop.

'When a stranger approached Mitchell, he eagerly began to touch some part of his body, commonly taking hold of his arm, which he held near his nose; and after two or three strong inspirations through his nostrils, appeared decided in his opinion. If it happened to be unfavourable, he suddenly went to a distance with the appearance of disgust; if favourable, he showed a disposition to become more intimate, and expressed by his countenance more or less satisfaction.'" 187.

Mr. Mayo proceeds to observe, that with human beings the faculty of perception is of slow growth, and that this is not owing to the general imperfection of the infant's mind, he concludes from the history of the couched patient related by Cheselden. That patient was born blind; when sight was given at adult age, he thought at first that all objects which he saw touched his eye, "as what he felt touched his skin." But this is by no means an analogous case. This gentleman had been educated, if we venture to use the expression, in the sense of *touch*—it was by touch only that he knew the more obvious qualities of the material world—this was, so far as vision was concerned, a false education—and can it be wondered at if experience was necessary to convince him of the inapplicability of the sense he had to a totally novel order of impressions.

But in animals, says Mr. Mayo, the faculty of perception is wonderfully perfect at first and at once, and he quotes, from Dr. Smith, the familiar instances of chickens, the young of the partridge and the grouse, the young of several sorts of animals, &c.

It appears to us that this is by no means so general a law as has been stated. Some animals are born more capable of independent existence than others. The blind kitten is little, if at all, better off than the puling babe. Who that has observed a nestling can pretend that the helpless, naked bird, was born with its instinct perfect and at once! It is notoriously not the fact. Nay, the perceptions of animals are only perfected by experience. The kitten that, prompted by its instinct, pursues with watchful activity the paper dangling from the schoolboy's hand, shuns such unprofitable and vain pursuit when matured into the cat. The cat that, for the first time, sees its image in the mirror, seeks or shuns its imaginary fellow; but a little experience makes it also wise, and, if it cannot reason philosophically on mirrors, it evinces a practical knowledge of the delusion. We are quite convinced that the more this matter of instinct is examined, the more will it appear that too much is attributed to it. That the human being is at its birth, and for some time afterwards, the most helpless of animals, we are willing to allow, but that the gulf between the reputed perfect instinct of the young of other animals, and its nascent reason, is so wide as is pretended, we are far, very far, from being convinced. In short, we look upon the unconditional expression of this assertion as unphilosophical and savouring somewhat of a vulgar fallacy.

But what is instinct? Take the following observations of Mr. Mayo.

"Upon referring to the ordinary operations of our own minds, volition appears to take place whenever we anticipate a greater degree of gratification or advantage from exerting than from repressing it. We know by experience the prompt influence of the will over our muscular frame: we are able to conjecture with more or less certainty the consequences of different voluntary actions: and we *will*, with a general or precise anticipation of what the result will be, and in order to obtain it. A hungry person knows that the food he prepares to eat will gratify his appetite: a drowning person hopes that his cries will bring people to his assistance. But there are instances in human beings in which intelligent motives cannot be assigned for voluntary actions. The infant at the breast, or struggling when first plunged into water, employs muscular efforts for its sustenance or preservation, no less *voluntary* than those which the school-boy makes when draining his orange, or the exhausted swimmer when he calls for help. But in the infant, the motive which leads to the voluntary effort, is not the anticipation of pleasure or advantage, but a spontaneous tendency, a blind inclination, an instinct.

Instinct then appears to consist in a natural tendency to execute certain voluntary movements, without any previous conception of the object they are calculated to attain, upon the occurrence of particular sensations or states of inward feeling. This account of instinct corresponds very nearly with the popular meaning of the term. The modifications of this property, as I have described it, are especially characteristic of human and brute intelligence: in man they are subdued and subservient to reason; in animals they greatly surpass in vigour and influence the faint glimmerings of reason which they exhibit, and in some instances curiously rival in their effects the most elaborate results of human thought." 190.

Mr. Mayo observes, that it may be denied that instinctive actions are voluntary, because we retain no consciousness of having exerted the will at the time of their performance. To this Mr. M. objects, that actions generally allowed to be voluntary may, when so frequently repeated as to constitute habit, be unconsciously performed. Mr. M. criticises Sir Charles Bell's theory of the respiratory or superadded system of nerves, and observes, with reference to the portio dura, that it is both a voluntary nerve and one of instinctive action. Voluntary actions may be substituted for instinctive ones; thus we may alter, for a time, our rate of breathing, mimic the expression of passions, and so forth. The conclusion, says Mr. Mayo, towards which the preceding arguments tend, has the additional advantage of being intrinsically more philosophical than that to which it is opposed; in classing instinct as a motive to the will, it is supported by analogy; while that which represents it as a principle equivalent at once both to motives and volition, disregards all analogy.

That the impulse which we denominate instinct does act, in a great many instances, through the medium of volition, cannot be disputed. But we are not convinced of the universal truth of Mr. Mayo's conclusion. Under the influence of the passions, which are instinctive, the voluntary muscles are not alone affected, but muscles and organs on which the will exercises no power are excited also. The first effect of fear is to check the circulation—the next to hurry the terrified individual backwards. So general is the law, that Bichat, as is well known, has actually, and no doubt wrongly, placed the seat of the passions in the breast. Is the instinctive impulse to void the urine or the fæces, or is the sigh, an act of volition? We think not. In short, it appears to us that instinct operates through both the voluntary and involuntary muscles, and whatever the instinctive impulse may be, wherever situated, or however obtained, it is something above the will, and acting upon it, in common with parts and functions with which the will has no relation. We have as good right to affirm that instinct regulates the organic system as the volition, for the secretions, nay, all the functions of organic life, are modified and controlled by it. If, then, the instinct be considered as a motive to the will, it must be considered as a motive to the nervous system of organic life also. We need scarcely add, that these considerations do not inform us what instinct is.

"I shall now quote one or two examples of the instincts of animals, which may contribute to persuade the reader that instinctive actions are voluntary, and which will at the same time bring us to another disputable question, as to where instinct ends and reason begins. 'On dissecting,' says Galen, 'a goat great with young, I found a brisk embryon, and having detached it from the matrix, and snatched it away before it saw its dam, I brought it into a room, where there were many vessels, some filled with wine, others with oil, some with honey, others with milk or some other liquor, and in others there were grains and fruits. We first observed the young animal get upon its feet and walk; then it shook itself, and afterwards scratched its side with one of its feet; then we saw it smelling to every one of these things that were set in the room, and when it had smelt to them all, it drank up the milk.' What is this but an instance of sensation occasioning a blind impulse to a determinate course of voluntary action." 193.

But suppose that the goat had lapped the oil or wine. Would not its stomach have rejected the unnatural food? Now we have no right to select the first of the checks against self-injury placed by Nature, as alone constituting instinct; and the circumstance of the stomach refusing to digest, what, if digested, would be prejudicial, would not be an instance of a sensation occasioning a blind impulse to a determinate course of voluntary action. The young animal probably preferred the milk, because it afforded most pleasure to its sense of smell or sight, and perhaps it is thus through the medium of the senses, that instinct is in many cases made by the Creator to act. But instinct often far transcends this.

"If a hive of bees be this year in possession of a queen duly fertilized, and consequently sure the next season of a succession of males, all the drones, towards the approach of winter, are massacred by the workers with the most unrelenting ferocity. To this seemingly cruel course, they are doubtless impelled by an imperious instinct: and as it is regularly followed in every hive thus circumstanced, it would seem at the first view to be an impulse as intimately connected with the organization and very existence of the workers, and as incapable of change as that which leads them to build cells or to store up honey. But this is far from being the case. However certain the doom of the drones this autumn if the hive be furnished with a duly fertilized queen, their undisturbed existence over the winter is equally sure if the hive have lost its sovereign, or her impregnation have been so retarded as to make a succession of males in the spring doubtful. In such a hive the workers do not destroy a single drone, though the hottest persecution rages in all the hives around them.*'

Now how are we to explain this difference of conduct? Are we to suppose that the bees know and reason upon this alteration in the circumstances of their community—that they infer the possibility of their entire extinction if the whole male stock were destroyed when without a queen—and that thus influenced by a wise policy they restrain the fury they would otherwise have exercised? This would be at once to make them not only gifted with reason, but endowed with a power of looking before and after, and a command over the strongest natural propensities, superior to what is expected in a similar case even from a society of men; and it is obviously unwarrantable. The more probable supposition is, that here again the conduct of the animal blindly follows an impulse originating from impressions on the senses: in other words, that a new instinct is developed, suited to the extraordinary situation in which the community stands, leading the work-

---

* Kirby and Spence's Entomology, vol. ii. p. 454.

ers now to regard with kindness the drones, for whom otherwise they would have felt the most violent aversion." 194.

It is evident that the latter supposition merely amounts to this, that the cause is instinct. The final intention is sufficiently clear, the efficient and immediate agency perfectly unknown. To believe that such foresight and precaution depend upon actual reflection, would argue such a degree of reason in these creatures as few can make up their minds to admit. But animals display what cannot but be considered more than instinct, more than a blind impulse, born with them, and perfect at its birth.

" It is evident that in animals, as in human beings, impressions once traced upon the mind may recur to it. Whoever has observed a dog during its sleep prick its ears and whine, must be persuaded that the animal dreams: in other words, it has the faculty of *conceiving* former objects of sense.

It is not less certain that animals have memory: this is shewn in the power of personal recognition which they evince.

The principle of imitation again exists in various degrees in animals. This principle is one that modifies instinct. A bird untaught will practise from instinct the song of its kind; but placed under circumstances where it hears another song exclusively, the young bird learns the notes in place of its proper song.

Animals display a principle analogous to the association of ideas, and that no less in a wild state than when tamed. The wariness which wild animals acquire with age is evidently attributable to this principle, and the education of domesticated animals is founded entirely upon it.

Animals certainly possess a power of attentive observation. When you have thrown a ball two or three times in succession for a dog to fetch, if you repeat the gesture of throwing, but retain the ball in your hand, the animal starts headlong after the object you have feigned to throw; but it quickly discovers the deceit: and now, when you would repeat the deception, the animal carefully watches whether the gesture of throwing be real or a feint, before it starts upon its course.

But the most remarkable illustrations of this faculty in animals, or rather perhaps of correct observation combined with surprising accuracy of recollection, are to be found in those cases in which animals have made their way home by a route they had never before pursued. That a dog should retrace its exact path for a hundred miles by the sense of smell, or that a carrier pigeon, even with the acute vision of a bird, should see its way towards home, are things sufficiently wonderful; but that an animal should discover its path home without any conceivable guide but *the remembrance of the direction* in which it has been brought, can scarcely be believed, even where its occurrence is perfectly attested. The following, which I extract from Messrs. Kirby and Spence's Entomology, is the most marvellous instance of the kind which I have read of.

' In March, 1816, an ass, the property of Capt. Dundas, then at Malta, was shipped on board the Ister frigate, Capt. Forrest, bound from Gibraltar for that island. The vessel having struck on some sands at Point de Gat, at some distance from the shore, the ass was thrown overboard to give it a chance of swimming to land—a poor one, for the sea was running so high, that a boat which left the ship was lost. A few days afterwards, when the gates of Gibraltar were opened in the morning, the ass presented itself for admittance, and proceeded to the stable of Mr. Weeks, a merchant, which he had formerly occupied, to the no small surprise of this gentleman, who imagined that from some accident the animal had never been shipped on board the Ister. On the return of this vessel to repair, the mystery was explained; and it turned out, that Valiante (as the ass was called) had not only swam safely to shore, but without guide, compass, or travelling map, had found its way from the Point de Gat to Gibraltar, a distance of more than two hundred miles, through a mountainous and intricate country, intersected by streams, which he had never traversed before, and in so short a period that he could not have made one false turn.'" 196.

But in other and in common instances, animals display the nearest approach to human reason. They acquire sagacity with age, that is, they are capable of profiting by experience. Examples of this must be familiar to all. The following singular fact is quoted from Dr. Fleming. The hooded crow of Zetland, when feeding on the testaceous mollusca, is able to break some of the tenderer kinds by means of its bill, aided in some cases by beating them against a stone; but as some of the larger shells, such as the buckie and the whelk, cannot be broken by such means, it employs another method, by which, in consequence of applying foreign power, it accomplishes its object: seizing the shell with its claws, it mounts up into the air, and then loosing its hold, causes the shell to fall among stones (in preference to the sand, the water, or the soil on the ground) that it may be broken, and give easier access to the contained animal. Should the first attempt fail, a second or a third are tried, with this difference, that the crow rises higher in the air, in order to increase the power of the fall, and more effectually remove the barrier to the contained morsel.

Mr. Mayo observes that, notwithstanding the capability for improvement, the contrast is still decided between the most sagacious animal, and man under the most unfavourable circumstances. In illustration of this he inserts Professor Glennes' very interesting account of a poor lad born blind and deaf. There is doubtless a broad gap between man and every other animal, but it is not because man possesses that of which every other animal is deprived—reason; but because he is gifted with a greater quantity than they. Define reason how we will, view it, consider it in what manner we may, it seems impossible to deny its existence in brutes. It certainly is not true that they possess only instinct, if by instinct be meant a faculty not improvable; and if instinct be improvable, in what does it differ from reason? The mind of brutes differs from that of man only in the degree of its rationality, and from the lowest in the scale upwards we discern an improving series. That man infinitely excels the brute there can be no question, nor need we wonder when we look at the immense development of his brain, and the general perfection of his nervous system. Suppose that the lower animals had been gifted only with instinct; a faculty at once perfect and susceptible of no further extension. Memory would have been of no service to them, because it would not have tempted them to seek former gratifications or avoid former dangers; when placed in new circumstances they could scarcely have accommodated themselves to them, and thus they would have been deprived of the benefit both of experience and of the power of accommodation to new relations. In short, to have given the lower animals a mind not susceptible of a certain amount of reflection and of improvement, would have been a mistake, which the all-wise Creator would never have committed, though many zealous and pious, nay, and able persons, have attributed it to Him; and to have given them more would have been inconsistent with the obvious plan of the creation and the mental supremacy of man.

Mr. Mayo proceeds, and in a very interesting manner, to enumerate the principles of human character, or of the human mind. He supposes the mature individual to have a rapid but distinct view of the façade of a building, for instance, King's College. From this one sensation he shews how perception—conception, or the power of calling up the scene again—the association of ideas—attention—imagination—comparison—judgment, may spring. Or the individual might ask himself, is it true that such or such an architect designed this building, or are the objects for which it was erected good? Mr. M. introduces here an analysis of truth, or of evidence, which we are tempted to extract. He observes that, positive or absolute truth may be said to be that which would command assent if all the evidence were produced and understood. Practically speaking, the evidence which determines belief, belongs to four classes, which are the following—

"A. Certain truths may be called intuitive, which whether shaped or not as distinct propositions, spontaneously arise in our minds, or are involved in all our reflections; and are of such a nature that to doubt them for an instant is impossible. Of this description are the following.

1. The belief which we entertain of the reality of those mental affections, of which we are conscious.

2. Our belief in the evidence of memory: in other words, our belief, when we remember to have experienced an emotion or impression before, that we really have experienced something similar on a former occasion. The strongest illustration which I can select of the conviction attending the evidence of memory, is our belief in our moral or personal identity; every man remembers feelings excited in him at a very early age by one incident or another; and recognizing on the evidence of memory their affinity to his present character, is sure that he who went through such an adventure is the same person who reflects upon it now.

3. The belief, that every change must have an efficient cause.

4. The belief that duration and space are without limits.

5. To many persons the evidence of perception appears of this description. The belief in the existence of an external material world bears indeed a close affinity to intuitive truth, inasmuch as it arises spontaneously in our minds, and cannot be seriously questioned by a rational understanding. Yet it must be admitted, that such is the constitution of our nature, that we can conceive it *possible,* that matter has no existence, and that our waking sensations, like those which we seem to experience in a dream, flow from some other cause than external material impressions.

B. Another class of truths comprise those which rest upon experience and analogy. When we have observed that certain consequences have uniformly followed certain antecedents, we are led by the constitution of our nature to expect the recurrence of those consequences, whenever we see their usual forerunners: thus, when we see the sun go down, we entertain no doubt that it will rise on the following morning; and so on in regard to the general order of nature; although it is evidently possible that that order may be interrupted to-morrow.

But the most remarkable illustration of the force which *may* belong to this kind of evidence, is to be found in the argument which the study of nature affords in proof of the existence of a God. All bodies, of which we can trace the history, that have a structure adapted to determinate purposes, have been contrived by intelligent beings. We therefore conclude analogically, when we meet with bodies of the origin of which we are ignorant, yet which have a structure and disposition adapted to determinate ends, that such instances likewise are the result of design, and have been produced by an intelligent cause. But Nature through all her reign exhibits to the most superficial, as well as to the profoundest observer, the most refined and sagacious adaptation of means to important ends. Nature then, we are compelled to believe, is but Art—the work of immeasurable wisdom and power.

A third instance of the conviction which flows from experience and analogy is, our reliance on testimony. The present instance serves remarkably to show the various shades of belief, which may attach to the kind of evidence which we are now considering. Who doubts that Socrates and our Saviour lived and died? and who believes the rumour of the day during the season of popular agitation? A child that has never been deceived, believes implicitly every assertion made to it. A statesman professes a distrust of history, upon his personal knowledge that it is sometimes falsified.

C. Mathematical truths are acknowledged to rest upon evidence so convincing, as to claim exclusively the title of *demonstration.* On what does the conclusiveness of demonstration depend? Or what is the nature of each step in mathematical reasoning? The proof in each instance amounts to this only: that the point in question is shown to be identical with one already admitted.

The certainty which belongs to syllogistic reasoning is of the same description. *Syllogistic proof* consists in shewing that the circumstance you would predicate of an individual has been already granted to belong to the genus.

*Induction* is the method employed in the discovery of physical causes. By means of an induction of well-chosen or well-contrived instances, the common and essential conditions in an entire class of phenomena are rendered apparent. Our belief in a law established by induction, arises from that law being but another and more general expression for what has been already shown to occur in every conceivable case that has admitted of investigation.

D. *Theoretical or circumstantial evidence,* is that which wins our belief by the concurrence of many probabilities towards one conclusion. The mind appears capable of receiving a collective impression from many single impressions. Every one knows how, in a fictitious tale, the feeling of horror excited by some harrowing scene is gradually heightened and wrought up by the skilful accumulation of incident upon incident. Thus in circumstantial evidence, each fresh probability augments the sum of our belief. There are many instances in which the degree of probability of an event admits of being stated numerically: in these the force of cumulative proof may be said to admit of mathematical demonstration." 203.

Mr. Mayo proceeds to describe Taste, and briefly alludes to Diversities of Talent and of Disposition, and enumerates those affections which form the active principles of our nature. Nothing can be more calculated to display the absurdity of that philosophy which regards the mind as a whole, a something half matter and half essence, of we know not what description or proportions, than the following conclusion at which some reasoners have arrived.

"All exercise of talent implies invention; from wit, which consists in presenting certain classes of ideas in novel and unexpected relations,—to poetry or design, in which new and just combinations of thought or conception are produced,—to philosophical genius, which is shown in creating or bringing together instances through which inductive truth may be elicited. Talent therefore of every kind works by the same processes; and thence it has been said, that difference of talent depends only upon the difference in the classes of associations upon which it operates, and thus that its particular direction in individual instances may possibly be accidental." 206.

It is right to observe that Mr. Mayo thinks it probably more just to look at the subject of, or the direction of talent as distinguishing, and its operations as subordinate, than to view the operations of the mind as the principal element, and the subject as an accident. When, undivested of the prejudices of the closet, we look at men as they are, we find them born with mental differences as striking as those which are observed in their corporeal frames. Under the same circumstances their talents, their tastes, their dispositions differ, and it is as philosophical to believe that the varieties of their complexions, or corporeal proportions, are the produce of some post-natal accident, as the equally marked varieties of their minds. But there is one fact which of itself conclusively demolishes the notion to which we have alluded. Parents with a tendency to insanity produce children with the same tendency. Now if the manifestations of mind did not depend on the qualities of organization, why should this be? Hereditary predisposition to disease is a common and an intelligible property of organized structures, but that the mind, "a something, nothing," should be generated lame and halting and impotent, is past comprehension, and defies belief. If then the *manifestation* of mind depends on organization, it follows, of course, that, organization being demonstrably different in different individuals, the phenomena of mind must differ also, which in fact they do.

It would almost be a waste of time, and certainly unbecoming in us, to prove that individuals are born with different mental capacities. Set a philosopher, who believes that peculiarities of talent depend on accidental circumstances, to educate a dozen Mozarts, or Homers, or Napoleons, or Newtons, and see how he would succeed. Let him take the material in its most ductile state—let him fashion the minds of his dozen neophytes from puling infancy upwards, and see how the experiment would succeed. He and his disciples would probably become denizens of Laputa.

"But what then is the mind, the possessor of these powers, the subject of these numerous affections and shades of humour? All that we know of it, is contained in the brief history of its affections, and the laws of their recurrence.

But is it, as the materialist imagines, the result of a particular combination of material atoms? Do thought and feeling flow from a change in organized structure, as music from the vibration of a string? Or is their subject something, which is essentially independent of, and may survive the dissolution of the corporeal frame?

Our knowledge upon this matter is contained in a small compass. In the first place, we can imagine that mind may exist without matter; there is no contradiction involved in supposing each material element of our frame destroyed, yet the distinct recollection of all we have done and suffered and enjoyed remaining. 2. It is utterly impossible for us to conceive how matter can produce mental phenomena. 3. We are in possession of the fact, that while our body changes, our mental identity remains. It is difficult to avoid concluding from these premises, that the human mind is something superadded upon and temporarily united to our living bodies, not a series of affections resulting from their material structure." 208.

We have shewn that brutes have something mentally in common with us, and it is acknowledged that we have instinctive feelings and passions in common with brutes. The mind, on Mr. Mayo's own shewing, is a composite thing; it is made up of capacities of thought and reflection, or the power of combining in many ways the impressions it has received, and of appetites and principles which it seems to bring with it, rather than to obtain from its intercourse with the world. Let us take then some of the attributes of the mind, or rather some of those phenomena, the aggregate of which we call mind, and let us see to what Mr. Mayo's argument will lead.

Memory, we suppose, is a mental phenomenon. Now memory is shared with man by "the worm that crawleth." There is scarcely a creature that can be supposed altogether deficient in it, and some possess it in a very remarkable degree. If memory then be a quality of the mind, and if it do not depend upon organization, on what does it depend in the dog, or the horse, or in animals lower and viler than they? If it be utterly impossible to conceive how matter can produce mental phenomena, then we cannot of course conceive that matter has produced this mental phenomenon in those inferior creatures, and we, of consequence, necessitated to grant to them "a something superadded upon and temporarily united to their living bodies." What that something is we leave Mr. Mayo to determine.

Gratitude is an attribute of the human mind of no mean character. Mr. Mayo therefore cannot well conceive how such can be the produce of matter. But gratitude and affection for his master are qualities displayed by the dog in no slight degree, and it is just as difficult to imagine the gratitude of the beast the result and function of material organization, as that of the man.

We conceive then that, if Mr. Mayo's argument is worth any thing, it is this—that the lower animals have souls as well as man. Whether Mr. M. does actually hold this Pythagorean doctrine we cannot say, but certainly his reasoning admits of the reduction we have mentioned.

How mental phenomena are produced is a question beyond human power to solve. But this is certain, that in proportion as the brain is developed in the ascending scale of organization, in that proportion is the mind improved and perfected. That this is correct as a general statement is as certain as any demonstrated truth, though some exceptions may apparently or really exist. Concussion or compression of the brain destroy thought, partial lesions occasion partial affections of it. There is doubtless, then, an intimate connexion between cerebral development and mental, and whether there be something beyond the brain, or whatever may be the case, the brain is certainly the *organ* of the mind, and mental phenomena are its functions. If it be said that this doctrine is materialism, we can only reply that we are bound to endeavour to arrive at truth, whatever may be the consequences. Materialism, however, in this sense, is not opposed to revealed religion. Well-meaning persons injure the cause of religion, when they aim at mixing it up with philosophical questions of this nature. The creation of man was long antecedent to the revelation of religion, and it cannot be expected that his constitution should furnish proofs of it. If there is nothing in the material organization of man that is absolutely contradictory to Revelation, that is all that we need ask. That there is no such contradiction in the dependence of the manifestation of mind on matter, appears to us to be very clear. We know not what the soul is; we know not that mind may not be dissevered from matter, and made as independent of it in another state as it seems dependent on it in this; in short, materialism, in the sense in which we have taken and take it, neither makes nor mars a creed, and we fly for consolation and assurance to the only hand whence we can receive it, that of the divine Founder of our Faith.

Mr. Mayo proceeds to consider the phenomena of sleep, dreaming, somnambulism, and spectral illusions. The remarks on dreaming are very interesting. He observes, in reference to the state of the pupil in those who sleep, that if awakened by opening the eyelids under a strong light, the pupil, for the first second or two, is seen to be extraordinarily contracted, which may be presumed to be its usual state during sleep. We have often examined the pupils of sleepers, and we have usually found them dilated. If the lids were opened under a strong light the pupil contracted, but even then it shewed a greater tendency to dilatation than the pupil of a waking person. Mr. Mayo believes that a certain degree of consciousness remains during sleep. We think this cannot be doubted, and the degree of consciousness is less in proportion as the sleep is *deeper*, or more approaching to a lethargic character. In reference to dreaming Mr. Mayo observes,—

"But the phenomena of dreaming are not always confined to the spontaneous suggestions of the fancy; occasionally the mind seems to bend itself during sleep to an examination of the impressions which occupy it. It must have happened to every one during a dream to have suspected that he was dreaming, and after a process of deliberate reflection to have become satisfied that he was awake.

It was the opinion of Dugald Stewart, that in sleep 'the will loses its influence over those faculties of the mind, and those members of the body, which during our waking hours are subjected to its authority.*' In the remarks which I have made upon the phenomena of consciousness, I have employed the term will to signify exclusively that affection of the mind, which is the immediate cause of muscular action: that *this* influence is not in every case suspended during sleep, appears evident upon the fact already adverted to, that many animals sleep in postures which require a sustained muscular effort. But we seem to exercise a voluntary power likewise over the affections of the mind: let us examine, before resuming the preceding inquiry, whether the latter influence be suspended during sleep. The faculty by which we direct the mind at pleasure to one train or mode of thought or to another, is essentially unlike that by which we produce a series of voluntary movements. Under ordinary circumstances we are indeed equally led to either,—to analyse for instance an affection of consciousness, or to strip the shell from a filbert,—by the gratification it promises; but while in the one case the effect we desire is attained directly and instantaneously,—we will and the muscles act,—in the former the effort consists in fixing the attention upon a subject of inquiry, and patiently observing the bearing of every thought, which presents itself, upon the point before us. In producing a muscular effort, we will a physical change, and it instantly ensues; in an effort of thought, we but confine the mind to a definite tract, expecting that our established habits of association will bring us to the conclusion we wish.

Now it appears from an instance of dreaming already mentioned, that the mind can during sleep set on foot an analytical inquiry, and may compare its different impressions in order to arrive at a conclusion respecting their nature,—an operation as voluntary, if the expression be applicable, as any which the mind exhibits in its waking state." 211.

We confess that we are not altogether convinced of the reality of the distinction that Mr. Mayo has drawn, between volition determining a muscular action, and that exercise of the will which may perhaps be fairly styled attention, and which directs the mind to any particular train of thought. That the muscle, in the one instance, acts at once and definitely, whilst, in the other,

* "Stewart's Philosophy of the Human Mind, vol. i, p. 330."

the train of thought which we will is not a speedy and determinate effect, seems to prove a difference between muscular action and thought, not a difference between that which commands the exercise of either.

It appears to us that, as sleep is not death, it would be equally absurd to suppose the functions of the brain annihilated during its continuance, as the functions of other parts or organs of the body. Sleep is a state of comparative, not of absolute quietude—of relative, not total incapability of receiving impressions through the senses. A slight noise makes no impression; a louder one does excite sensation, and awakes. It is equally so with sight, and smell, and touch. Slightly pinch the skin of the sleeper, and, by an exercise of the voluntary muscles, he withdraws the limb, but does not awake. Pinch him more severely, and the sensation is not only sufficient to produce enough consciousness to will the contraction of the muscle, but it awakes him, that is, the consciousness becomes complete. Sleep is not an uniform state, but is different in degree in different individuals, or at different times in the same individual. That difference of degree seems merely to mark a greater or less amount of consciousness in the brain—a greater or less sensibility to impressions through the senses.

Dreaming is manifestly an excitation of the brain, by former sensations or by present impressions, at a time when its higher attributes or functions of attention, comparison, judgment, are little, if at all exercised. It should be recollected that our sensations or impressions are not merely those which we derive from the external senses, but are called up by our organic sympathies or instinctive feelings. Thus in the state of perfect waking, and at a time when the mind is most finely strung, the presence of some superabundant acid in the stomach, dyspepsia, however produced, will mar its exquisite attunement, and prostrate the reason into gloomy hypochondriacism. The valiant man, under this poor sympathy, will become for the time a coward—the philosopher a fool. As our impressions, when awake, are twofold, so are they twofold when asleep; but there is this difference, that the senses being closed, or nearly closed, against their ingress, memory asserts her almost undivided reign, and the dream is of things gone by. The organic functions still go on, and the brain is liable to be affected by its sympathies or connexions with them. The dyspepsia, or the dyspnœa from affections of the heart, which produce in the waking individual hypochondriacism, or sensations of distress, give rise in the sleeper to impressions which his half-conscious mind cannot so properly appreciate, and hence the exaggerated nightmare and the frightful dream. Such a state has been well described by Scott, in his description of the anxious night spent by Roderick Dhu.

> "In fitful dreams the image rose
> Of varied perils and pains and woes;
> Now his steed founders in the brake:
> Now sinks his barge beneath the lake:
> Now, leader of a broken host,
> His banner falls, his honour's lost."

No disquisitions that we have ever read have offered a satisfactory solution of the immediate cause of sleep, or dreams. These are the ultimate facts that have ever baffled, and will ever baffle human reason, and speculations directed towards them are profitless waste of thought and time.

But when we carefully analyse phenomena, the legitimate task of philosophy, we think we discern, and that not very obscurely, some of the links in their production, though we know not the immediate and efficient agency.

The last part of this subject to which we shall advert, is Mr. Mayo's comparison of the brain in man and brutes. As we think Mr. M. has displayed some considerable fallacies, we will give his reasoning entire.

" Let us now turn to the physiological conclusions which are suggested by a comparison of the brains of vertebral animals of different orders with each other and with that of man.

Whenever our knowledge of any branch of natural philosophy is sufficient to enable us to study with success the uses of a part of the animal frame, we invariably discover the most exact adaptation of the physical structure to the office of the part. In the form and disposition of the bones, for instance, or in the structure of the eye, we find more and more occasion for wonder at the perfect art displayed, in proportion as we more deeply study mechanics and the nature and properties of light. It is impossible for us therefore to doubt, that in the brain there is the same exquisite fitness to the purposes for which it is designed. And as experiment and observation lead us to think, that the brain is the organ through which the mind influences and is influenced by the body, we naturally conclude that its whole structure has a direct and immediate relation to the endowments and workings of the mind. The composition of the brain is doubtless as exactly suited to the phenomena of thought and feeling, as the structure of the eye to the properties of light. What inquiry is likely to prove more interesting, than to trace the relation which exists between an improving mental nature and its appropriate bodily instrument?

It does not appear that an increase in the absolute weight of the brain confers a superiority in mental endowments. Were this the case, the intellects of the whale and of the elephant should excel the rational nature of man. Neither does the relative weight of the brain to the whole body appear the measure of mental superiority. The weight of the human brain is but $\frac{1}{35}$ of the frame; while that of a canary bird is $\frac{1}{14}$. Nor, in conjunction with parity of form and structure even, does this relation appear of any value. The eagle is probably as sagacious as the canary-bird; but the weight of its brain is but $\frac{1}{260}$ of its entire weight.

We may next inquire, whether an increasing number and complication in the parts of the brain is essentially connected with improved mental functions. The first instances which occur to the mind are in favour of the affirmative of this supposition. It may be inferred from their docility and surprising capability of receiving instruction, that birds have higher mental endowments than fish; and accordingly, in place of the nodules of the fishes' brain, which are scarcely more than tubercles to originate nerves, birds possess an ample cerebrum and cerebellum. But in pursuing this argument, if we compare, on the other hand, the brain of birds with those of alligators and tortoises, we find no striking difference or physical superiority in the former over the latter; yet in mental development the tortoise and alligator are probably much nearer to fish than to birds. The instantia crucis, however, upon this question, is found in the comparison of the brain of the cetaceous mammalia with the human brain on the one hand, and with that of fish on the opposite.

The cetaceous mammalia have brains which, besides being of large size, are nearly as complicated as those of human beings; they might therefore be expected, if the opinion which I am combating were true, to manifest a remarkable and distinguishing degree of sagacity. Endowed with a brain approaching nearly in complexity and relative size to that of man, the dolphin should resemble in his habits one of the transformed personages in eastern fable, who continued to betray under a brute disguise their human endowments. Something there should be very marked in his deportment which should stamp his essential diversity from the fishes, in whose general mould he is cast. His habits too, not shunning human society, render him especially open to observation; and the class of men who have the constant opportunity of watching his gambols in the deep are famed for their credulity, and delight to believe in the mermaid, the sea-snake, and the craken. Yet the mariner sees nothing in the porpesse or the dolphin but a fish, nor distinguishes him, except by his unwieldy bulk, from the shoal of herrings he pursues. The dolphin shews, in truth, no sagacity or instinct above the carp, or the trout, or the salmon. It is probable even that the latter, which have but the poorest rudiment of a brain, greatly exceed him in cunning and sagacity.

I am afraid that the instance which I have last adduced, is sufficient to overthrow most of the received opinions respecting the relation of the size and shape and organization of the brain to mental development; nor is it easy to find a resting place for conjecture upon this subject.

Is there, we may ask, any ascertained relation between a single division of the enkephalon, and any class of mental affections? It is often asserted, that some ratio exists between the size of the cerebellum and the force of the sexual appetite: yet, how opposite to this conclusion are the most obvious facts. The cerebellum of frogs, which are remarkable for their salacity, is so small, that its existence has been disputed. Fish, on the other hand, have the cerebellum of great size. And in mammalia, in which the sense of smell certainly contributes principally to excite the sexual appetite, the olfactory nerve, which is of prodigious size, instead of having any visible connexion with the cerebellum, appears to be an actual production of the cerebrum.

Or can any relation be traced between the development of the brains of animals and the caste of their other organs? We naturally suspect that such may be found, when we observe, that animals so unlike in their habits (at least as far as regards their mode of progression) as the frog and the snake, have brains precisely similar. We are led to think, by such instances, that the formation of the brain may have a greater reference to the physical character of the general frame than to the mind. Yet the brain of the ornithorhyncus, as it is delineated by Meckel, resembles not the brains of reptiles and birds, to which by its generative organs the animal seems allied, but the brains of mammalia.

The brains of monkeys have a strong general resemblance to the human brain. This must surely refer to the resemblance of their physical organs to those of man, not to their sagacity: the dog should else have a brain shaped like that of the monkey.

It is a common opinion, that the front of the brain is the seat of the intellectual faculties: yet in monkeys and in man the back part of the brain is that which has the largest relative size. The sheep, on the other hand, has an ample front to its brain, a large intellectual region, according to the phrenological theory, whilst its instinct of attachment to its young has a poor locality in its moderate posterior cerebral lobe.

M. Magendie makes the curious remark, that the brains of adult human beings exceed those of aged persons fifteen per cent. in specific gravity.

Has nothing then been discovered to mark an essential superiority in the brain of man? The question must, I believe, be answered in the negative. No physical condition, distinguishing the human brain from that of animals, and therefore fitting it to co-operate with a rational soul, has as yet been ascertained, or even plausibly conjectured, to exist. We are as yet free to suppose, that a mental nature of a higher caste, may possibly work with materials as gross as those which are suited to the instincts and small sagacity of brutes; or that some delicate difference of organization, finer than we yet have the means of testing, and independent of the relative size and volume of the brain, may be the material cause, especially qualifying the human brain to be the seat of reason." 252.

Mr. Mayo proves too much, for if his arguments lead to any conclusion it is to this, that man's superior reason is not owing to his brain at all—a rather startling announcement. But let us examine Mr. Mayo's positions. Man's brain is not distinguished by its absolute weight, says he, because the brain of the whale and the elephant are heavier—nor by its relative weight, because that is but 1-35th of the weight of his frame, whilst the brain of the canary bird is 1-14th of the weight of its frame—nor by its complication, because the cetaceous mammalia have brains which are not only large, but *nearly* as complicated. Now before we deal more particularly with these statements, let us just employ analogically the same argument, and so display its *general* futility. We will suppose that the question is as to the physical superiority of man and animals. Mr. Mayo, we imagine, would say, that man is physically not superior to animals, and he would urge, that the nightingale has a more melodious voice, the eagle a keener eye, the hound a finer scent, many animals a quicker ear, many too a softer skin or a more alluring surface, the lion more strength, the antelope more agility, in short, he might shew that in almost every physical peculiarity some animal or other possessed the advantage. But what would be the straight-forward reply to such a line of argument? True, these creatures you have instanced excel the human being each in some particular, but is there any animal that possesses the *general* physical endowments that man does, is there one that combines so many qualities in so much perfection?

Mr. Mayo may select the particular instances he has done, with reference to cranial development, but we put it to Mr. Mayo's candour to declare whether the brain of any animal that he has examined, or that any other person has examined, will bear comparison for one instant with that of man, in *all* its qualities of size, arrangement, complication. Who that looks at a brute's brain is not instantly struck with the fact, that there is little more than pertains to the senses; at least, that the chief proportions of their brains are those which are connected with the nerves? Where do we see that immense superimposed mass of hemisphere, that would seem preposterously and uselessly placed in the human skull if it were not connected with human intellect? Mr. Mayo instances the brain of the sheep, as possessing an ample front, a large intellectual region. Mr. Mayo's sheep must be very different from any we have seen, or he would not venture to compare its dwarfish cerebrum, the development of which above the thalami appears contemptible, with the overpowering mass of human cerebral matter. Let any one unprejudiced person place the brain of the sheep and of the man side by side, and rise with impressions adverse to the connexion between cerebral and mental development, if he can.

Here we fear we must pause. It may appear singular that, after speaking in such terms of praise of Mr. Mayo's work, we should have criticised so freely. But in fact we have only selected points for criticism, and those of a confessedly debateable character. The great body of the work must pass unchallenged, though there are some doctrines advocated to which we cannot yield undivided assent, such for instance, as that of transudation in the living tissue. We conclude, however, by recommending the volume in the most cordial manner to the public, and especially to students. It is a work which does honour to the school from which it emanates. We trust that in the criticisms in which we have indulged, we have displayed no dogmatic spirit. We are fully aware that the truth is not necessarily on our side, and that we are very likely to be wrong in the opinions we entertain. The public are the best judges of conflicting sentiments, and if the public think that Mr. Mayo's opinions on the points at issue are more correct than are ours, we shall bow very willingly to the decision.

